# Comparing Images from Near-Infrared Light Reflection and Bitewing Radiography to Detect Proximal Caries in Primary Teeth

**DOI:** 10.3390/children11121455

**Published:** 2024-11-28

**Authors:** Aviv Shmueli, Avia Fux-Noy, Esti Davidovich, Diana Ram, Moti Moskovitz

**Affiliations:** Department of Pediatric Dentistry, Faculty of Dental Medicine, Hebrew University, Hadassah Medical Center, P.O. Box 12272, Jerusalem 91120, Israel; avia.fux@mail.huji.ac.il (A.F.-N.); esti@dr-st.co.il (E.D.); dianar@ekmd.huji.ac.il (D.R.); motim@ekmd.huji.ac.il (M.M.)

**Keywords:** NIRI, X-ray, children, detection, proximal caries

## Abstract

Objectives: The present prospective study aimed to compare near-infrared light reflection (NIRI) and bitewing radiographs (BWR) images to detect proximal caries in primary teeth. Methods: 71 children underwent routine BWR, and scans were performed using an intra-oral scanner (iTero Element 5D, Align Technology, Tempe, AZ, USA), including a near-infrared light source (850 nm) and sensor. Five specialist pediatric dentists examined the NIRI and BWR images. Results: The average participant age was 7.8 years. A total of 1004 proximal surfaces of primary molars and canines were examined, 209 carious lesions were detected on BWR, and 227 on NIRI. Comparison between all carious lesions detected on BWR and NIRI: Sensitivity (53.6%); Specificity (85.5%); Positive Predictive Value (PPV) (49.3%); Negative Predictive Value (NPV) (87.5%). Comparison between carious lesions involving the DEJ detected on BWR and at any level in NIRI: Sensitivity (61%); Specificity (83.4%), PPV (36.6%); NPV (93.2%). Comparison between enamel-only carious lesions detected on BWR and all lesions detected using NIRI: Sensitivity (44.8%); Specificity (85.5%); PPV (20.7%); NPV (94.8%). Conclusions: No additional diagnostic information can be gleaned from BWR if initial caries lesions in the enamel are not detected by clinical examination or in images from a NIRI scanner, making BWR unnecessary.

## 1. Introduction

Clinical examination or direct visual examination enables the detection of many pre-cavitated lesions and cavities in primary teeth. However, proximal caries in primary molars and canines cannot be detected in this manner. In the primary dentition, there are usually interdental spaces until age 4, when the contact points between the primary molars are established. This increases the potential to develop proximal caries and necessitates detection. In contrast to the progression of proximal caries in permanent teeth that can take a few years, in primary teeth this process is rapid, and caries can approach the pulp tissue within months, making early detection and treatment essential. Ticotsky et al. found that approximately 0.8 years were required for a carious lesion to progress from the outer enamel to the dentin-enamel junction and an additional 1.4 years to reach the inner part of the dentine [1]. The morphology of primary teeth, including thinner enamel and dentine, lower degrees of mineralization as well as wider dentinal tubule lumens, explains the rapid progression of caries. The wide proximal contact area of primary molars allows greater accumulation of cariogenic microorganisms compared to the permanent tooth, and also plays a role [2]. When not detected and treated, caries can progress to the pulpal tissues and cause pain, swelling and sometimes the early loss of the primary tooth, which may cause a lack of space for the permanent successor and a need for orthodontic intervention. In their systematic review and meta-analysis, Uribe et al. reported a worldwide prevalence of 48% for caries in preschool children. In Africa the prevalence was 30%, in the Americas 48%, in Asia 52%, in Europe 43% and in Oceania 82% [3]. Bitewing radiographs (BWR) enable the effective detection of decayed surfaces but have low specificity regarding sound surfaces [4]. For decades, BWR have been the standard imaging technique for detecting early proximal lesions. However, this traditional method has its limitations. Carious lesions that have progressed into the dentine and reached a threshold where restoration is necessary are seen in BWR. However, early-stage caries may be missed [5]. Early detection of caries can prevent the need for restoration by stabilizing or remineralizing the tooth surface, thereby maximizing natural tooth tissue retention.

In 2009 Subka et al. assessed the validity and reproducibility of four methods of proximal caries detection in primary molars. They concluded that radiographic examination is superior at the stage of dentine caries [6].

Radiation protection practices aim to minimize the risks of cancer induction and heritable effects and eliminate tissue reaction risks [7]. Exposure to ionizing radiation might influence the genetic material in human cells and cause oncogenic alterations. Therefore, patient shielding during dentomaxillofacial radiography is recommended [7], and intervals between X-ray examinations should be carefully considered. Early caries may present as a non-cavitated carious lesion (NCCL). Although the enamel surface remains intact, these lesions can reach the dentine. Imaging is required to monitor these early lesions, and the risks of using ionizing radiation for imaging limit the use of X-rays for this purpose. Consequently, there have been many studies using new technologies aiming to accurately detect NCCLs. Reviews by Serban et al. and Janjic et al. concluded that when it comes to clinical practice and proximal NCCLs, the diagnostic accuracy of the emerging technologies does not allow them to replace conventional radiography [8,9]. The most promising alternative methods are based on the optical properties of enamel, including transillumination using near-infrared light. Intact enamel appears transparent when near-infrared light is passed through it. However, carious lesions scatter and partially reflect the light, leading to a visual distinction between sound and carious enamel. Devices for detecting proximal caries using near-infrared light reflection (NIRI) have been developed. When utilizing this technology, teeth are exposed to NIRI, and the reflection is captured and displayed as a grayscale image. In this image, healthy enamel, which is transparent to light, appears dark, while carious lesions, which scatter and reflect the near-infrared light, appear brighter than the surrounding enamel [10,11].

The iTero Element 5D (Align Technology, Ltd.) is an intraoral optical scanner used for acquiring digital impressions. The system digitally captures the 3D geometry and color of intra-oral dental structures with a proprietary optical, non-contact, focus detection technique that uses Class 1 laser technology (these radiation levels are incapable of causing damage according to the American National Standards Institute). As mentioned above, NIRI is a non-ionizing imaging technology that leverages differences in scattering and absorption of near-infrared light that depend on the degree of tooth mineralization. The iTero Element 5D has near-infrared illumination capabilities which enable the detection of proximal caries at various stages, ranging from initial enamel caries to established caries reaching the DEJ. The device provides an image of the teeth without using ionizing radiation.

This study aims to compare the diagnostic quality of the iTero Element 5D system to routine bitewing radiographs in detecting primary interproximal carious lesions in deciduous molars and canines above the gum line.

## 2. Materials and Methods

This prospective cohort study was conducted in The Department of Pediatric Dentistry, Hadassah Medical Center, Faculty of Dental Medicine, The Hebrew University of Jerusalem, Israel.

1. Ethical considerations: The study protocol was approved by the Institutional Human Subjects Ethics Committee of Hadassah Medical Organization IRB, Jerusalem. All procedures followed the ethical standards of the institutional and national research committee (HMO0351-22). The study protocol was enrolled and can be accessed in full at clinicaltrials.gov (NCT05792631). A detailed information sheet in simple nontechnical language was provided and parents/guardians signed an informed consent form before participation. No compensation was given to the participants.
2. Inclusion criteria: age 4–9 years, scheduled visit for bilateral BWR or bilateral BWR performed within 2 weeks of the study visit. Exclusion criteria: diagnosis of epilepsy, allergy to latex, plastic or dental/oral health products, underwent dental treatment following their most recent BWR. All participants were given a unique identification number linked to their data. De-identified data sets were used for analysis.

During the initial visit, consent, screening, enrollment, and imaging were performed. A follow-up call took place one month later where the patient records were reviewed and any additional clinical information following the trial visit was recorded e.g., if a treatment plan was made.

Routine clinical and diagnostic methods for the diagnosis of caries such as a thorough visual examination and bitewing radiographs (BWR) were used in this study. *** Specifically, the radiographs were taken using phosphoric plates with a standard sticker/tab, no holders were used. The imaging software for the radiographs was MediaDent version 8.2—Dental Practice Management and Digital Radiography Software, as used for all patients treated in The Department of Pediatric Dentistry.

Bite Wing Radiographs (BWR) including the complete posterior dentition of the subject were taken, (unless they were performed within 14 days of the study visit). Additionally, the maxillary and mandibular arches of all subjects were scanned using the iTero Element 5D system. **** The specialists performing the scan used well-known behavior management techniques such as tell-show-do and modeling.

The lesions in the BWR and iTero Element 5D images were graded according to ADA staging guidelines [12]. The outcomes were recorded on Caries Evaluation Forms and data was entered into an Excel spreadsheet. Each BWR and NIRI scan was examined by 5 experienced specialist pediatric dentists.

Evaluators had no experience evaluating NIRI images before the study. All participants were trained. Calibration was achieved by giving each examiner 20 anonymous images to evaluate. Based on this, inter-evaluator reliability was measured, and inter-examiner Kapa was 0.832.

Sample size was calculated using the sample size equation for comparing paired nominal data by using McNemar’s test [13]. Based on relevant literature [13] and the results of previous trials, we assumed a non-inferiority margin of 5%.

(1) $$n=\frac{{\left[z_{\alpha }\sqrt{p_{01}+p_{10}}+z_{\beta }\sqrt{p_{01}+p_{10}-{\left(p_{01}-p_{10}\right)}^{2}}\right]}^{2}}{{\left(p_{10}-p_{01}\right)}^{2}}$$

Similar formulae can be obtained for the one-sided non-inferiority test by substituting $z_{\alpha /2}$, $p_{10}$ with $z_{\alpha }$ and $p_{10}+M$:

(2) $$n=\frac{{\left[z_{\alpha }\sqrt{p_{01}+p_{10}+M}+z_{\beta }\sqrt{p_{01}+p_{10}+M-{\left(p_{01}-p_{10}-M\right)}^{2}}\right]}^{2}}{{\left(p_{10}+M-p_{01}\right)}^{2}}$$

where significance level α=0.05, power 1−β=0.8, non-inferiority margin *M* = 5%, assumed detection rate $p_{01}=0.22\%$, $p_{10}=2.46\%$.

The minimum sample size required was 89 surfaces. Assuming the drop-off rate to be 30%, an additional 39 surfaces were recruited, so the total number of surfaces needed was 128. By assuming a minimum of 2 naïve IP surfaces per pediatric patient, we determined the minimum required patient and site count was 70 patients at one trial site.

### Statistical Analysis

The extent of agreement between the NIRI and BWR readings was assessed by calculating the Kappa measurement of agreement. The McNemar test for paired data was applied to test the difference between the NIRI and BWR readings. Sensitivity, Specificity, Positive Predicted Value (PPV), and Negative Predicted Value (NPV) were calculated for the NIRI readings, regarding the BWR reading as the Gold Standard. The statistical test applied was two-tailed, and a *p*-value of 0.05 or less was considered statistically significant.

## 3. Results

A total of 71 children, 43 males and 28 females, with an average age of 7.8 years, (median 7.9 years) and SD of 1.82 participated. Of the total, 62 had a mixed dentition and 9 had a primary dentition.

Of the 1704 proximal surfaces of the participants, findings from 1004 proximal surfaces were included in the examination. Carious lesions were found on 209 surfaces. Lesions were classified as degree 1 and 2 for initial enamel caries, and degree 3 when the lesion involved the dentine. Treated teeth were removed from the sample.

**Figure 1:**
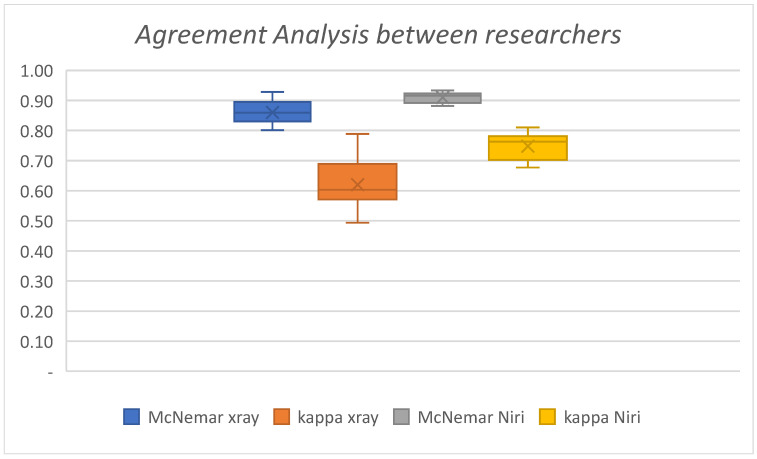
Box-plot Agreement Analysis between examiners.

1. Agreement Analysis between specialists in both BWR and NIRI
  A compatibility analysis was conducted between the examiners for the results from both BWR and NIRI images. The McNemar Test and Kappa were utilized for statistical evaluation, as seen in Figure 1 and Table 1.
2. Following initial analysis, the final grade for each surface was calculated using the majority decision of the five examiners from the BWR and NIRI images. Subsequently, three comparisons were made:
  A. Comparison between any dental caries lesions detected on BWR (ground truth) to NIRI (Table 2): Sensitivity (Yellow): 53.6% Specificity (Orange): 85.5% Positive Predictive Value (PPV) (Blue): 49.3% Negative Predictive Value (NPV) (Green): 87.5%.
  B. Comparison between caries lesions involving the DEJ detected on BWR to detection at any level in NIRI (Table 3): Sensitivity (Yellow): 61% Specificity (Orange): 83.4% PPV (Blue): 36.6% NPV (Green): 93.2%.
  C. Comparison between lesions limited to enamel only on BWR versus any lesion detected with NIRI. Lesions involving the CEJ were excluded, leaving 862 surfaces for analysis (Table 4):

Sensitivity (Yellow): 44.8% Specificity (Orange): 85.5% PPV (Blue): 20.7% NPV (Green): 94.8%.

## 4. Discussion

The present study compared the diagnostic quality of images from the iTero Element 5D to intra-oral bitewing radiographs (BWR) for detecting primary interproximal carious lesions in deciduous molars and canines above the gum line. We found that the images from the iTero device were better for detecting initial lesions, but not superior for detecting cavities that progressed into the dentine. This finding may reduce the number of X-rays prescribed to children during routine dental check-ups if they can be scanned using a NIRI scanner.

Caries progress rapidly in primary teeth. The BWR study by Tickotsky et al. found that it took approximately 0.8 years for a proximal carious lesion to progress from the outer enamel to the dentine-enamel junction and an additional 1.4 years for it to reach the inner part of the dentine [1]. This highlights the importance of early detection in the primary and mixed dentition. Proximal lesions, especially in the early stages, may not be noticed during a visual examination. The principal method for detecting proximal dental caries is through BWR.

In recent years, parents and pediatric dentists have expressed significant concern about exposing children to ionizing radiation. Birant et al. found that parents of children who had never undergone dental radiography had a more negative attitude towards dental radiographs than those who have had dental radiographs [14]. In addition to concerns regarding radiation, intra-oral X-rays present a challenge for some children. Sometimes, parents need to stay with their child in the treatment room or hold their child while X-rays are taken, and this causes some inconvenience.

The recommendations of the American Academy of Pediatric Dentistry for prescribing intra-oral X-rays for children with a primary or transitional (mixed) dentition are based on caries risk assessment. For patients with high dental caries risks the recommendation is a BWR exam at 6–12-month intervals if proximal surfaces cannot be examined visually or with a probe. For patients at low risk of dental caries, BWR is recommended every 12–24 months if proximal surfaces cannot be visually or physically examined [15].

The European Academy of Pediatric Dentistry recommends that the intervals between intra-oral radiographic examinations should be based on the individual’s age and the most advanced proximal caries lesion identified during the latest radiographic caries assessment. Shorter intervals may be recommended for high caries-risk patients with extensive lesions, while longer intervals can be chosen for less caries-active individuals [16]. In addition, they suggest considering X-ray-free alternatives before dental radiographs are prescribed, but only with an understanding of their diagnostic efficacy [17,18].

Agreement between examiners was evaluated using two statistical tests, the McNemar and Kappa tests. Both tests showed greater agreement when reading images from NIRI than BWR. BWR was treated as the gold standard for proximal caries detection. Even though our examiners are experts at reading BWR, there was still variability in caries detection. This is probably because some examiners have a more conservative attitude than others. Nevertheless, agreement between examiners was quite high.

The intra-oral optical scanner iTero Element 5D (Align Technology, Ltd.) used in this study showed a Specificity of 85.5% with a positive predictive value of 20.7% and negative predictive value of 94.8% in comparison to BWR, for lesions limited to the enamel. When no cavities are detected during a clinical examination of a patient with low caries risk, BWR can be replaced by near-infrared light reflection scanning, based on the clinical judgment of the dentist. This is because the likelihood of detecting early cavities in bitewing X-rays is only 5%.

Metzger et al. found that near-infrared light reflection was more sensitive than BWR in detecting early enamel lesions and comparable to bitewing radiography in detecting lesions that involved the DEJ [19]. Our findings are partly consistent with Metzger et al. We examined the proximal surfaces of primary molars and canines in children with primary and mixed dentitions, whereas Metzger et al. only studied permanent teeth. The characteristics of primary teeth may influence the results of near-infrared light reflection scanning. Enamel wear is common in primary teeth, and wear facets on the mesial or distal aspects of the occlusal surface might be interpreted as caries in images from near-infrared light reflection scanning. This issue can be resolved by thorough clinical examination and experience.

The trend toward minimally invasive dentistry, especially in pediatric dentistry must be accompanied by a reliable means for the early detection of initial carious lesions. The findings of this study indicate that near-infrared light reflection technology may serve as a simple and convenient tool for scanning pediatric dental patients with no clinical evidence of caries. The scanner is easy to use and comfortable for the patient, and the scanning procedure is rapid, and importantly, does not use ionizing radiation. As for all diagnostic tools, there is a learning curve for handling the scanner and interpreting the images.

Future studies might show higher specificity and sensitivity values as clinicians gain experience with the near-infrared light reflection tool.

## 5. Conclusions

No additional diagnostic information can be gleaned from BWR if initial caries lesions in the enamel are not detected by clinical examination or in images from a NIRI scanner, making BWR unnecessary.

## 6. Clinical Importance

Currently, NIRI cannot be used instead of BWR, but it can serve as an additional tool for pediatric dentists. In children with low caries risk and no clinical signs of caries, clinicians might consider using NIRI technology instead of BWR to avoid ionizing radiation for diagnostic purposes.

### Study Limitations

This study had some limitations that warrant discussion. The cohort included 71 patients; future investigations with more participants may reveal more significant results and validate the findings of the current investigation. Furthermore, the novel nature of NIRI in pediatric dentistry meant that the examiners had much more experience reading BWR than NIRI images, and some of the discrepancies may be due to the implementation of this new diagnostic tool. As clinicians gain experience, their ability to interpret the images and accurately detect proximal caries in primary teeth will improve, as will the significance of our findings.

## Figures and Tables

**Table 1:** Agreement between examiners regarding findings from BWR and NIRI images.

McNemar						Kappa				
	Mean	SD	Range	LCL	UCL	Mean	SD	Range	LCL	UCL
BWR	0.86	0.04	0.13	0.84	0.88	0.62	0.08	0.3	0.57	0.67
NIRI	0.91	0.02	0.05	0.9	0.92	0.74	0.045	0.13	0.72	0.78

**Table 2:** Comparison between any detections in BWR and any detections in NIRI.

			NIRI Overall, Any Detections		
			No	Yes	Total
BWR overall any detections	No	Count	680	115	795
		Percentage within BWR overall, any detections	85.5%	14.5%	100%
		Percentage within NIRI overall, any detections	87.5%	50.7%	79.2%
	Yes	Count	97	112	209
		Percentage within BWR overall, any detections	46.4%	53.6%	100%
		Percentage within NIRI overall, any detections	12.5%	49.3%	20.8%
Total		Count	777	227	1004
		Percentage within BWR overall, any detections	77.4%	22.6%	100%
		Percentage within NIRI overall, any detections	100%	100%	100%

**Table 3:** Comparison between lesions involving the DEJ detected on BWR compared to any detections in NIRI:.

			NIRI Overall, Any Detections		Total
			No	Yes	
BWR overall, any Dentine detections	No	Count	724	144	868
		Percentage within BWR overall, any Dentine detections	83.4%	16.6%	100%
		Percentage within NIRI overall, any Dentine detections	93.2%	63.4%	86.5%
	Yes	Count	53	83	136
		Percentage within BWR overall, any Dentine detections	39%	61%	100%
		Percentage within NIRI overall, any Dentine detections	6.8%	36.6%	13.5%
Total		Count	777	227	1004
		Percentage within BWR overall, any Dentine detections	77.4%	22.6%	100%
		Percentage within NIRI overall, any Dentine detections	100%	100%	100%

**Table 4:** Comparison between lesions limited to enamel only on BWR versus any lesion detected with NIRI.

			NIRI Overall, Any Detections		Total
			No	Yes	
Any teeth with Enamel involvement BWR	No	Count	680	115	795
		Percentage within any teeth with Enamel involvement BWR	85.5%	14.5%	100%
		Percentage within NIRI any detections	94.8%	79.3%	92.2%
	Yes	Count	37	30	67
		Percentage within any teeth with Enamel involvement BWR	55.2%	44.8%	100%
		Percentage within NIRI any detections	5.2%	20.7%	7.8%
Total		Count	717	145	862
		Percentage within any teeth with Enamel involvement BWR	83.2%	16.8%	100%
		Percentage within NIRI any detections	100%	100%	100%

## Data Availability

The data that support the findings of this study are available from the corresponding author upon reasonable request.
